# Supplementary material for: Are carbon emissions trading and green financial instruments synergistic? -Comprehensive quantitative research based on content analysis
Source: PLoS One. 2024 Mar 7;19(3):e0298601. doi: 10.1371/journal.pone.0298601 (PMC10919601; doi:10.1371/journal.pone.0298601)
Supplement: S1 Data — (ZIP) [file pone.0298601.s001.zip › Data/地级市Z_F工作报告.zip!/╔┬╬≈/═¡┤¿╩╨/└┤╫╘╣1⁄2╓┌║┼í╛┬φ┐╦╩2╛▌═°í┐_2016.docx]

各位代表：

　　现在，我代表市人民政府向大会报告工作，请予审议，并请市政协委员和列席人员提出意见。

　　一、市十五届人大六次会议确定任务和“十二五”规划完成情况

　　2015年，面对错综复杂的宏观环境，特别是经济下行压力加大的不利局面，全市上下认真贯彻落实中省决策部署，主动适应经济发展新常态，攻坚克难，锐意进取，较好地完成了市十五届人大六次会议确定的目标任务，主要经济指标增速高于全省平均水平。

　　（一）有效应对下行压力，经济持续稳定增长。在传统支柱产业遭受重创的情况下，我们主动作为，精准发力，经济运行缓中趋稳、稳中有进、进中有好。实现生产总值324.54亿元，增长8.8%，高于全省0.8个百分点；地方财政收入23.11亿元，同口径增长13.7%，高于全省1.6个百分点；城乡居民收入29560元、10058元，分别增长8.5%、9.7%。居民消费价格上涨1.2%。开展了项目观摩、国企进铜等活动，100个市级重点项目完成投资182.7亿元，占年度投资任务的121.8%，新开工项目36个，18个项目建成投运。全社会固定资产投资完成383.98亿元，增长17.2%，高于全省9.2个百分点。在全省率先出台了工业稳增长12条措施，积极务实地组织稳煤救铝增电销水泥，铜铝公司120台电解槽复产，4大支柱产业对工业经济增长贡献率70.4%。新增规模以上工业企业22户，工业品产销率97.3%。规模以上工业增加值165.76亿元，增长8.5%，高于全省1.5个百分点。

　　（二）转型发展有序推进，产业结构趋于优化。装备制造、食品加工、医药保健等接续产业实现增加值12.54亿元，增长10.5%。董家河园区列为国家循环化改造试点，工业园区产值占全市工业产值的55.2%。关闭煤矿6处，淘汰落后产能335万吨。阿庄风电、天辰光伏项目实现并网发电。实施重点旅游项目39个，完成投资12.7亿元，申河、哭泉、金锁等乡村旅游逐步升温。接待游客1213万人次，旅游总收入65.61亿元，分别增长22.2%、22.4%。社会消费品零售总额增长13.9%，高于全省2.8个百分点，增速全省第一。服务业增加值增长11.2%，高于全省1.6个百分点，增速全省第二。粮食总产24.11万吨，实现“十二连丰”。周陵园区列为国家农业科技园区、产业示范基地。水果总产73.02万吨，增长5.7%，建成万亩现代果业示范区3个，全国“一村一品”经验交流会在我市召开。宜君玉米、耀州黄芩获国家农产品地理标志认证。

　　（三）扎实开展四城联创，城市品位不断提升。我市成功创建为国家卫生城市，在全国爱国卫生工作会议上交流发言。徒步街巷现场管理步入常态。新区九州东道、西环线北段建成通车。南市区集中供热面积22万平方米。铜川植物园建成开放。新增城市绿地27万平方米。宜君县创建为省级生态园林县城。黄堡重点示范镇和照金、陈炉文化旅游名镇建设加快，完成投资5.9亿元。2个部级、20个市级美丽乡村和10个新型农村社区建设进展顺利。

　　（四）加大环境治理力度，生态建设成效明显。坚持控烟减煤抑尘增绿，多措并举治污降霾。完成了华能照金电厂超低排放改造，拆除燃煤锅炉223台，新建燃气锅炉141台，淘汰黄标车1511辆，新增天然气用户9000户。实施了3个矿山地质环境治理项目，植树造林13.26万亩，治理水土流失201.68平方公里。万元生产总值能耗下降6.6%，降幅高于全省3.3个百分点。四项主要污染物减排任务超额完成，全年空气质量优良天数269天，较上年增加40天。繁育出铜川籍朱鹮16只，娄勤俭省长在第三届中美省州长论坛上，推介了铜川转型发展、改善生态环境的成效。

　　（五）改革开放扎实推进，发展活力不断增强。45项重点改革任务全面完成。取消了非行政许可审批，实行涉企收费目录清单制度。新登记市场主体8504户，增长46.8%，非公经济占生产总值比重50.2%，提高1.6个百分点。在全省率先建成四级便民服务网络。基本药物制度实现县以下公办医疗机构全覆盖。与美国约巴琳达市缔结为友好城市。成功举办了第三届中国孙思邈中医药文化节和“一带一路”国家中医合作研讨会。招商引资到位资金144.87亿元。外贸进出口总额2282万美元。

　　（六）切实保障改善民生，群众有更多获得感。财政民生支出73.01亿元，较上年增加6.26亿元。城镇新增就业9772人，登记失业率3.34%。为8.22万城乡低保对象发放保障金2.15亿元。建成保障房13240套。解决了3.26万农村居民安全饮水问题。1.8万农村贫困人口脱贫，王益区整体脱贫。朝阳实验小学建成招生，耀州区（新区）、宜君县、王益区通过国家义务教育发展基本均衡区（县）评估认定和省级“双高双普”评估验收，中小学教育群众满意度排名全省第一。全市17个乡镇食品药品监管所通过省级示范所验收。建成社区服务项目26个。安装广播电视户户通设备6875套，实施体育惠民工程74个，实现残疾人辅助器具适配全覆盖。全市生产经营性事故死亡人数占省控指标的57.89%，未发生较大及以上事故。社会治安和信访工作不断加强，社会大局总体稳定。

　　（七）职能转变稳步推进，政府建设得到加强。扎实开展“三严三实”专题教育，机关作风明显好转。完成“六五”普法目标任务。公开区县、市级部门预决算，“三公经费”较上年下降28.7%。市政府会议、文件分别下降15%和4%。1405名公民代表走进政府，60名公众代表列席政府会议，326名大学生到机关见习。办理人大代表建议106件、政协提案297件。“铜川发布”荣获陕西十大党政新闻发布微博第三位。市政府门户网站访问量4000万人次。办结人民网留言102条、“市长信箱”2165件。2015年度全市总体工作“两代表一委员”满意度测评全省第一，群众满意度测评全省第三。

　　过去一年，国防动员、双拥、统计、审计、民族、宗教、人防、气象、地震、档案等工作也都取得了新成绩。

　　各位代表，2015年主要目标任务的完成，标志着“十二五”圆满收官。五年来，全市人民团结奋进，埋头苦干，战胜诸多困难和挑战，较好地完成了“十二五”规划纲要确定的主要目标任务，全市经济社会发展站在了新的起点上，为在全省早日全面建成小康社会奠定了坚实基础。

　　——这五年，是我市经济实力明显增强的五年。全市经济发展换挡不失速，生产总值年均增长12.9%，高于全省1.8个百分点，人均生产总值3.84万元；固定资产投资累计完成1319.4亿元，年均增长26.8%；地方财政收入较“十一五”翻一番，年均增长10.9%；小康实现程度79%，五年提高了10.8个百分点。

　　——这五年，是我市转型发展步伐加快的五年。县区、园区、景区竞相发展，传统产业不断升级，接续产业发展势头较好，循环经济体系正在形成。照金红色旅游名镇建设成效显著，孙思邈中医药文化影响力不断提升，旅游人数和旅游总收入年均分别增长19.6%、44.3%。三次产业结构由7.6︰62︰30.4调整到7︰59.3︰33.7。全国转型城市绩效考核、节能减排财政政策综合示范考核，获得优秀格次。

　　——这五年，是我市城乡面貌变化较大的五年。“北市区—黄堡—董家河—耀州—新区—坡头”带状组团型城市空间格局初步形成。耀州区列为全省新型城镇化试点，照金、董家河、黄堡、陈炉、彭镇被确定为全国重点镇，孟家塬、陈坪、马咀、陈炉、广阳荣获全国“一村一品”示范村镇。全省居民幸福指数、城市宜居水平排名，我市分别位列第二、第三名。城镇化率63%。

　　——这五年，是我市发展保障能力稳步提升的五年。生态环境明显改善，先后荣获省级园林城市、全国绿化模范城市和国家卫生城市称号。万元生产总值能耗累计下降24.8%。延西高速铜川段、合凤高速铜旬段、耀旬二级公路建成通车。建成高速公路142公里、国省干线及县乡公路329.6公里、通村公路320公里，公路密度高于全国、全省平均水平。骨干电网全面建成。龙潭水库大坝成功截流。

　　——这五年，是我市群众得到实惠最多的五年。城镇新增就业7.39万人。7.6万农村贫困人口脱贫。在全省率先实现城乡居民社会养老保险一体化全覆盖。城镇基本医疗保险参保率、新农合参合率98%、99.4%。城乡居民收入年均增长13.2%、16%。城镇人均住房面积增加7.21平方米。

　　各位代表，这些成绩的取得，是省委、省政府坚强领导、关心支持的结果，是市委统揽全局、科学决策的结果，是市人大、市政协及社会各界监督支持的结果，更是全市人民咬定目标、苦干实干的结果，承载着历届市委、市政府打下的良好基础。在此，我代表市人民政府，向全市人民，向关心支持铜川发展的各界人士，表示崇高的敬意和衷心的感谢！

　　我们也清醒地认识到，我市发展还存在不少困难和问题，政府工作还存在一些不足。经济下行压力还在持续，接续产业规模小、占比低、发展慢，稳定经济增长、加快转型发展的任务艰巨；科技创新能力不强，经济外向度不高，资金、人才等要素集聚能力较弱；城市规划建设管理水平有待进一步提升，治污降霾、水污染防治任务艰巨；民生领域仍有不少群众不满意的地方，部分群众生活困难，脱贫攻坚任务较重；政府职能转变还不到位，一些政府工作人员责任担当和创新意识不强，不作为乱作为和不会为不善为的问题依然存在。对此，我们必须拿出事不避难的勇气，采取更加有力的举措破难题补短板，让全市人民感受到实实在在的变化和成效！

　　二、“十三五”发展思路和目标任务

　　“十三五”是我市全面建成小康社会、实现深度转型的决战决胜期，经济社会发展机遇与挑战并存。世界经济在深度调整中曲折复苏。我国发展步入新常态，经济发展长期向好的基本面没有变。中央、全省城市工作会议为加快新型城镇化指明了方向。我市发展思路更加清晰，发展环境不断改善，一些支撑全市转型发展的重大项目取得突破性进展，全市干部群众心齐气顺劲足，攻坚克难的底气和自信不断增强。这些都是我市更好更快发展的基础和条件。

　　供给侧结构性改革，从长远看对我市转型发展有利，但短期内对我市传统支柱产业会带来冲击。省上把煤炭开发的重点放在陕北和彬长地区，控制和压缩渭北地区的煤炭产能，我市煤炭产业收缩趋势将更加明显；国家下调燃煤发电上网电价，对我市电力产业发展带来一定影响；电解铝价格持续低位，铜铝公司维持生产困难重重；水泥市场竞争加剧，企业兼并重组将会成为主流。因此，全市经济社会发展正经历着改革转型的阵痛，机遇和挑战前所未有。我们要因势而谋，顺势而为，以新理念把握引领新常态，以新思路应对破解新挑战，以新状态开创发展新局面。

　　“十三五”全市经济社会发展的总体思路是：全面贯彻党的十八大和十八届三中、四中、五中全会精神，紧扣“五位一体”总体布局、“四个全面”战略布局和“五个扎实”的要求，以五大发展理念为引领，以在全省早日全面建成小康社会为目标，以深度转型、追赶超越为主线，突出“三重”（重要区域、重点工作、重大项目）抓“三区”（县区、园区、景区），大力实施文化引领、创新驱动、绿色发展、区域协同、共建共享、开放融合“六大战略”，积极推进新型工业体系构建、现代服务业多元发展、特色农业提质增效、文化引领全域旅游、基础设施保障、创新能力提升、新型城镇化推进、区域协调联动发展、脱贫攻坚及民生共建共享、美丽家园建设“十大工程”，奋力开创转型铜川、美丽铜川、健康铜川、富裕铜川建设新局面。

　　“十三五”的主要发展目标是：在提高发展质量和效益的前提下，生产总值年均增长9%左右，达到560亿元，人均超过1万美元；地方财政收入同口径年均增长12%左右，达到36亿元；城乡居民人均可支配收入年均增长9%左右，达到3万元，现行标准下农村贫困人口全部脱贫；深度转型成效全面显现，生态环境质量全面改善，社会治理能力和文明程度明显提高，在全省早日全面建成小康社会，“四个铜川”建设迈上更高水平。

　　坚持创新驱动，构建新型产业体系。创新是引领发展的第一动力。深入挖掘科技创新潜力，大力实施“互联网+”行动，构建具有铜川特色、体现创新引领的现代产业体系。用高新技术改造提升传统产业，促进煤炭就地转化，产业可持续发展能力显著增强。依托工业园区，促进科技创新，加快发展装备制造、新能源、食品、医药等接续产业。区域化布局、产业化带动、园区化示范、规模化经营，大力发展优质高效、特色生态农业，提高农业现代化水平。加快发展电子商务、现代物流、商贸流通、金融保险等现代服务业，到2020年服务业增加值占生产总值比重40%以上。搭建众创、众包、众扶、众筹新平台，聚合“四众”之力促“双创”。非公有制经济占生产总值比重达到55%以上。

　　坚持区域协同，推动城乡协调发展。协调是转型发展的内在要求。围绕促进渭北区域中心城市建设和西铜一体化发展，按照“南拓西展、北疏中控”的思路，加快构筑“一带贯南北、两心缀七区”的城市空间布局。统筹城市、建制镇、中心村协调发展，突出新区引领地位，推动新耀协同创新发展，促进北市区联动融合发展，扶持宜君绿色可持续发展，构建开放、包容、协同的区域发展新格局。推进智慧城市、海绵城市建设，以交通网络、水利设施、市政配套、智能电网建设为重点，完善城市功能。加快美丽乡村建设，提高公共服务均等化程度，推进农业转移人口市民化，城镇化率提高到70%。

　　坚持文化引领，全面提升城市品质。文化是铜川最为厚重、最珍贵的资源。深入挖掘历史文化内涵，大力发展文化旅游、健康养生等产业。全面建设照金香山、药王山、玉华宫、陈炉古镇四大景区，构建“一心两轴四区多点”的全域旅游新格局。坚持文旅融合，城市建设要融入历史文化基因，把景区打造成精品，把城市打造成景区。加快建设一批独具特色的乡村旅游模范村，打响乡村旅游品牌。大力发展智慧旅游，提升服务品质，建成1个5A级景区、3个4A级景区、10个3A级景区。

　　坚持绿色发展，推进美丽铜川建设。绿色富市，绿色惠民。坚持尊重自然、顺应自然、保护自然的生态文明理念，实施山水林田湖生态保护和修复工程，筑牢生态安全屏障。大力实施造林绿化，有效保护森林和湿地资源，提升区域生态承载能力，全市森林覆盖率达到48.5%，水土流失治理程度提高到65%。以改善环境质量为核心，强化环境综合治理，打好大气、水、土壤污染防治三大战役。持续实施治污降霾保卫蓝天行动，优化大气环境质量。加强水源地保护，加大漆水河、沮河、赵氏河水污染综合治理，改善水环境质量。开展土壤环境污染调查工作，完成土壤分级区划，建立健全土壤环境数据库。积极支持绿色清洁生产和消费，主要污染物排放强度指数全面达标，万元生产总值能耗降低12%。绿色低碳循环成为发展主基调，实现生产空间集约高效、生活空间宜居适度、生态空间山清水秀。

　　坚持开放发展，着力实现合作共赢。开放是繁荣发展的必由之路。抓住“一带一路”战略机遇，加强与沿线地区文化、旅游、经贸交流合作，把铜川建设成为特色鲜明的丝绸之路经济带重要节点城市。加强与西安海关的合作，做好外贸进出口企业服务保障工作。充分发挥我市资源循环利用、水泥、陶瓷和果业等方面的优势，坚持以企业为主体，实行市场化运作，推动优势产能、特色产品“走出去”。抓住中东部产业转移的机遇，精准招商，全面提高经济外向度。

　　坚持共享发展，增进城乡群众福祉。共享是发展的本质要求。更加重视保障和改善民生，把政府工作的成就感建立在市民的获得感上。实施积极的就业政策，城镇新增就业4万人以上。优化义务教育布局，加快学前教育发展，打造优质高中教育，加快发展现代职业教育。加强文化基础设施建设，扎实推进国家公共文化服务体系示范区创建工作。大力发展体育事业，实现村级农民体育健身工程全覆盖。完善城乡医疗卫生体系，优化医疗资源配置，深化医药卫生体制改革，提高全民健康水平。

　　各位代表，“十三五”规划凝聚着社会各方智慧，承载着全市人民的期盼。我们坚信，只要全市上下团结一心，坚定地干、扎实地干、精准地干，“十三五”宏伟蓝图必将变为美好现实！

　　三、2016年的主要工作任务

　　今年是实施“十三五”规划的开局之年。我们必须牢固树立和贯彻五大发展理念，坚持稳中求进、改革创新，认真落实去产能、去库存、去杠杆、降成本、补短板五大任务，围绕“三重三区”，突出抓好“两稳两优两增”（稳投资、稳产业，优结构、优环境，增就业、增收入），不断增强持续增长动力，实现新常态下全市经济社会更好发展，确保“十三五”开好头、起好步。

　　经济社会发展主要预期目标是：生产总值增长9%左右，规模以上工业增加值增长9%左右，地方财政收入同口径增长12%左右，全社会固定资产投资增长15%左右，城乡居民人均可支配收入分别增长9%、10%左右，居民消费价格涨幅控制在3%左右，城镇登记失业率控制在4%以内，万元生产总值能耗、主要污染物减排完成省上下达的指标。

　　（一）抓住关键稳增长，促进经济企稳回升

　　着力抓好项目建设。项目建设是稳增长的关键。在优化投资结构和质量的前提下，努力扩大有效投资规模。今年继续筛选一批市级重点项目，集中力量推进，力争完成投资160亿元以上。美鑫45万吨铝镁合金及自备电厂等22个项目建成投产投运，华能照金电厂二期及铁路专用线等43个项目开工建设，煤矿采空区光伏发电等25个前期项目取得实质进展。加强对央企进陕、国企进铜、中医药文化节等活动签约项目的跟踪服务，争取签约项目早日落地建设。下大气力优化投资环境，健全投资环境评价机制和社会监督体系，标本兼治解决影响投资环境的突出问题。加大服务重点项目建设“铜七条”的落实力度，夯实区县和园区主抓项目、市级部门服务项目的责任，坚持一线督导进度、及时协调服务、现场解决问题、定期观摩评比，全力保障项目按时间节点快速推进。

　　狠抓产业提质增效。工业是稳增长的核心。加强工业运行监测和调度，继续落实和完善工业稳增长的有关措施，强化服务保障工作，“一业一策”“一企一策”，确保支柱产业、骨干企业顺应发展、平稳增长。积极实施直供电试点，打造低电价区。鼓励煤炭、水泥、电力、铝业企业互动联保，拓宽煤炭、水泥销售渠道，积极争取将铝锭纳入全省有色金属收储范围，争取增发电指标。龙头带产业、产业兴园区，加快8个工业园区基础设施建设，提升园区承载能力，在产业项目引进上实现新突破，推动园区投入产出步入良性循环，力争工业园区经济增速高于全市2－3个百分点，全市新增规模以上工业企业10户以上。粮食总产20万吨以上。打响铜川果业品牌，加快果树试验站建设，将铜川苹果、大樱桃和宜君核桃创建成省著名商标，水果总产75万吨。抓好畜牧养殖和道地中药材规范化种植，创建1个省级、4个市级标准化畜牧养殖场。大力发展生产性服务业和现代服务业，全市服务业增加值增长11%以上。

　　努力扩大消费需求。消费是稳增长的基础。从严控制商品房项目审批，严格控制新建租赁型保障房，把重点放在商品房去库存、提高保障房入住率上。加快保障房小区配套设施建设，解决好群众入住“最后一公里”问题。通过市场化手段筹集保障性房源，推行政府购买棚改服务工作，实施棚户区改造8000户，棚户区改造货币化安置率不低于50%。大力发展商贸流通业，加快推进商业综合体、专业市场、特色商业街区建设，积极开展商品促销活动。落实带薪休假制度，扩大休闲、旅游、文化等体验性消费。鼓励发展中医药养生、健康调理、体育健身、老年照料护理等新型业态，促进医养结合，引导健康消费。完善农村流通网络体系，加快村级电子商务服务站建设，促进大宗农产品网上交易，扩大农村消费。社会消费品零售总额增长13%。

　　（二）定向施策优结构，推动产业转型升级

　　推进新型工业化。坚持工业强市、质量强市战略，推动工业从依赖资源、增量扩能为主转到调整存量、做优增量、提升质量上来。煤炭产业立足转化找出路、开新路，加快行业整合重组，重点引进实施深加工项目，推进煤炭清洁高效利用，重塑行业优势。水泥产业依托技改和管理提效益，控制生产规模，加强品牌建设，发展水泥制品，延伸产业链条，提高附加值。陶瓷产业以市场需求为导向，加强与知名陶瓷企业对接与合作，积极引进新技术、新工艺，提升工艺陶瓷、日用陶瓷、建筑陶瓷的市场竞争力。以创新引领装备制造业向中高端迈进，提升医药、食品行业核心竞争力，有序开发石油、油页岩等资源，积极发展光伏、风电等新能源，提高接续产业对经济的贡献份额。

　　促进旅游品牌化。推进全域旅游、全景铜川建设，继续实施重点旅游项目建设，完成投资10亿元以上。启动陕西智慧旅游大数据中心项目，实施智慧景区建设，加快照金景区、陈炉古镇核心区、药王山和玉华宫景前区建设，支持宜君县域旅游发展。按照A级景区标准，抓好申河、马咀、孟姜女故里、金锁石林、哭泉等乡村旅游发展，打响特色品牌。孙思邈纪念馆建成开放，推进王石凹工业遗址公园建设。实现3A级以上景区标准化公厕全覆盖。搞好旅游宣传营销，加大旅游产品开发。全年接待游客突破1320万人次，实现旅游总收入72亿元以上。

　　加快农业现代化。以结构性改革强农惠农，建设区县农村产权流转交易市场，加快农村资源资产化、股份化步伐。支持龙头企业通过直接投资、参股经营、签订长期合同等方式联农带农，引导大中专毕业生、返乡人员等领办合作社和家庭农场。全年培育龙头企业10户以上，培养新型职业农民300人。推进土地经营权有序流转，加快5个现代农业综合示范区和33个现代农业园区建设，完成投资1亿元以上。建成市级农业信息综合服务平台，完善区县农业信息服务网络，组建100名农技服务专家和50名乡土专家队伍，实现市县村网络信息服务互联互通。推进气象灾害监测体系建设，提高人工防雹作业能力。扩大政策性农业保险覆盖面，实现小麦、玉米保险全覆盖。

　　（三）坚持以人为核心，推进新型城镇化

　　强化城乡规划管控。切实转变城市发展方式，系统思维、集约发展，着力提高城市发展的持续性、宜居性。推进“多规合一”，做好城市总体规划修编，启动风景名胜区总体规划修编，完成全市“十三五”美丽乡村发展规划编制。树立“盆景城市”的理念，加强城市色彩、建筑风格管控，开展重要节点、街区城市设计。理顺规划管理体制，严肃查处各类违法违规建设行为。

　　完善城市服务功能。树立经营城市的理念，拓宽融资渠道，吸引社会资金参与城市建设。按照“先地下、后地上”和绿色低碳循环的理念，加快城市道路、供排水、垃圾处理等基础设施建设，建成华原西道、华夏南道，开工建设新区西环线、北环线部分路段。加快城市供水管网二期改扩建和供水提质改造工程建设，确保饮用水质达标，实施居民用水用电户表改造工程。南市区集中供热面积达到300万平方米以上。

　　提升城市治理能力。按照国家卫生城市标准，抓好国家卫生城市巩固提升工作，积极开展健康城市创建。结合建管职能分离，理顺城市管理体制，优化力量配备，推进综合执法，下移执法重心。开展城镇环境卫生综合整治活动，完善徒步街巷现场管理及巡查考核机制，实现主次干道机械化清扫、路面冲洗和道路保洁全覆盖。完善提高数字化城管系统，推进智慧城市建设。启动国家园林城市和省级生态园林城市创建，继续实施城市亮化、美化工程，展示动静相宜、昼夜变换的城市之美。创新城市社区治理，规范住宅小区物业管理。

　　提高新型城镇水平。积极做好新区创建国家级经济技术开发区基础工作。搞好耀州城乡统筹发展试点，加快推进宜君秀美山城建设。抓好黄堡重点示范镇和陈炉、照金文化旅游名镇建设，增强产业辐射带动和人口聚集能力。促进有能力在城镇稳定就业和生活的常住人口有序实现市民化。采取以点带面、市区县联动、部门协作的办法，整合资源、压茬推进，全面启动5个美丽乡村示范片区和60个美丽乡村创建村建设。

　　（四）硬法硬手治污染，着力优化生态环境

　　加大治污降霾力度。全面落实《环境保护法》《大气污染防治法》和铜川环保“铁十条”，以最严格的标准、最严格的措施、最严格的监管、最严格的问责，治污降霾保卫蓝天。精细化治理火力发电、水泥企业、非煤矿山等工业污染，限期拆除城市规划区和主要交通道路两侧废弃建（构）筑物，完成拆除场地的生态恢复。常态化联合执法治理道路、建筑工地扬尘，严查运输车辆超速、超载、抛洒行为。狠抓车辆尾气治理，鼓励使用新能源汽车，加大客运车辆使用清洁燃料力度。推广红旗社区网格化管理经验。每日公布各区县空气质量排名。强化科技治污，建设环保“天眼”工程，加强部门联防联控，确保空气质量持续好转。

　　持续加强生态建设。认真实施天然林保护、三北防护林建设、退耕还林等林业重点工程，突出抓好城市、园区、景区、道路、流域、庭院六大重点区域造林绿化，山上治本、身边增绿，植树造林10万亩。重视湿地保护和恢复，加快赵氏河、福地湖国家湿地公园建设。加快建设龙潭水库和印台后河沟水库，力争开工建设宜君王家坪水库。治理水土流失150平方公里。加强农村饮水工程建设和管护，保障农村饮水水质达标，解决2万人安全饮水问题。完成地质灾害避险搬迁293户1070人。开工建设漆水河、沮河城市段综合整治工程。

　　节约集约利用资源。做好节能减排财政政策综合示范工作，推进绿色低碳循环发展。落实能源和水资源消耗、建设用地等总量和强度双控行动，实施全民节能、节水、节地行动计划，抓好工业、建筑、交通、公共机构等重点领域节能。实施雨洪资源利用和中水回用工程，全市用水总量控制在1.45亿立方米以内。落实最严格的耕地保护和节约集约用地制度，严控增量、盘活存量、优化结构、提高效率，加大闲置土地处置力度，推进城镇低效用地再开发和工矿废弃地复垦。加快垃圾分类回收体系建设，促进资源再利用。

　　各位代表，绿色是万象之元、健康之基，绿水青山就是金山银山，以更大的力度，呵护好天蓝地绿水清的美好家园，让好山好水好空气成为铜川最靓丽的名片！

　　（五）改革开放求实效，增强发展新动能

　　推进供给侧结构性改革。精准研判煤炭、水泥市场变化，寻找新的产销平衡点，不能盲目扩大生产，稳妥化解过剩产能。稳妥处置“僵尸企业”，对停产半停产、连年亏损、资不抵债和靠政府补贴及银行贷款运转的企业，在安置好职工的前提下，坚决实行兼并重组、债务重组乃至破产清算。开展降低企业成本行动，紧跟中省政策，最大程度地降低企业交易、人工、物流、财务成本和税费、五险一金、电力价格等成本，多渠道为实体经济让利。利用人民银行扶贫开发金融服务工作联系点和“金惠工程”重点推广区的机遇，创新金融产品和服务，优化金融生态环境，提高政府性融资担保机构担保能力，引导金融机构加大对实体经济的支持力度。在公共服务、资源环境、基础设施等领域进一步创新投融资机制，全面推广政府和社会资本合作模式，充分发挥社会资本特别是民间资本的积极作用。

　　深化国企等领域改革。出台深化国资国企改革的实施意见和相关配套意见，大力推进国有企业整合与兼并重组。健全完善国有资产监管体制，实施市属企业国有资产统一监管、分类考核，推行市属国有企业外派监事制度，在市建设集团开展国有企业薪酬制度改革试点。认真落实我市加强财源建设的意见和激励考评办法，构建良性循环的财源建设新格局。加快财税体制改革，全面实施零基预算，将政府性债务全口径纳入预算管理，做好存量债务置换工作。深化“放管服”改革，加强事中事后监管，完善市场监管体制，落实行业、主管部门监管责任，增强放权的协同性、监管的有效性、服务的便利性。上半年完成市、区县政府“权力清单”“责任清单”编制工作，并向社会公布。营造支持中小微企业发展的政策、法制、市场环境，加快非公有制经济发展。完善镇村综合改革相关工作，分类推进事业单位改革，稳妥推进事业单位公车改革。全面实行居民用电、用水、用气阶梯价格。探索建立合理的容错机制，鼓励敢闯敢试，发挥基层和群众首创精神，激发改革创新活力。

　　抓好招商引资工作。围绕传统产业改造、接续产业培育，加大精准招商工作力度，大力推进产业链招商，争取在引进高新技术产业、战略性新兴产业、现代服务业等项目上有新突破。紧盯国内500强、行业领军企业等知名企业，瞄准高校、科研院所的科技成果，坚持选商选资、招商引智，广泛开展专业招商、定向招商活动。积极参加“西洽会暨丝博会”“陕粤港澳经济合作周”“农高会”等重大招商活动，力争在“招大引强”上有新突破。落实责任、优化环境、跟进服务，提高签约项目履约率和资金到位率。全年利用市域外资金150亿元以上。

　　积极扩大对外开放。统筹推进与“一带一路”沿线地区全方位交流合作，扩大向东向西开放。大力培育外贸骨干企业，精心打造铜川品牌，利用重要会展平台，扩大苹果、陶瓷等地产品出口。外贸进出口总额2520万美元。

　　（六）兜底补短强保障，共建共享惠民生

　　打好脱贫攻坚战。按照“五个一批”的要求，把片区攻坚与精准到户有机结合，精准产业、搬迁、生态、教育、社会兜底等脱贫措施，市、区县每年按不低于地方一般预算收入的2%安排专项扶贫资金，并确保每年增长不低于20%，各类惠民政策和涉及民生的专项转移支付资金向贫困地区和贫困群众倾斜。建立和落实扶贫工作任务清单、措施清单和责任清单，实行包村、包户、包人，促产业发展、促劳动就业、促农民增收的“三包三促”工作机制，确保2.1万贫困人口脱贫。

　　完善机制增就业。面对“去产能”可能带来的就业趋紧形势，实施更加积极的就业政策，健全就业创业服务体系，降低政策受惠门槛，激发大众创业、万众创新。抓好省级创业型城市创建工作，发挥“三个基地”示范带动功能，扎实做好就业困难高校毕业生、退役军人及“去产能”分流人员等重点群体就业托底工作。建设技能人才培训基地，开展适应市场需求的免费职业培训行动，提高劳动者创业就业技能。全年发放创业担保贷款2亿元，城镇新增就业8000人以上。

　　多渠道增加群众收入。健全企业职工工资决定和正常增长机制，促进职工工资合理增长。完善农民工工资支付保障制度。继续提高退休人员基本养老金待遇水平。健全税收、社会保障、转移支付等再分配调节机制，增加城乡居民的经营性收入、工资性收入、财产性收入和转移性收入，逐步缩小城乡、区域、行业、群体之间收入差距。

　　强化社会保障工作。全面实施机关事业单位养老保险制度改革。建立统一的城乡居民基本医疗保险制度，提高城乡居民基本医疗保险财政补助标准，全面实施城乡居民大病保险，健全重特大疾病救助制度。建成7个城市社区服务项目、14个农村社区服务中心。开工建设市老年服务中心，办好农村幸福院、社区日间照料中心。做好农村五保户、城市“三无”人员、高龄和失能老人、残疾人生活保障，关爱农村留守儿童、妇女和老人，支持志愿服务和慈善事业发展。

　　繁荣发展文化事业。大力弘扬和践行社会主义核心价值观，加强思想道德建设，持续放大“铜川好人”效应，在全社会营造崇德向善的浓厚氛围。传承照金精神，打响照金干部教育品牌。深入开展省级文明城市创建，提高城市文明程度和市民文明素质。抓好国家公共文化服务体系示范区创建，推进文化体育惠民工程，开工建设市文化体育基地，政府购买公共文化演出服务400场。举办市第三届文化艺术节，做好参加第十一届中国艺术节剧目演出和铜川分会场的组织工作。

　　提升公共服务水平。全面完成第二期学前教育三年行动计划，全面实施十三年免费教育，改善薄弱学校办学条件，提升基础教育办学水平。抓好印台区义务教育均衡发展和“双高双普”创建工作。资助400名家庭经济困难大学生，为6000名大学生提供生源地助学贷款。积极支持铜川职业技术学院规划建设，努力改善办学条件。深化县级公立医院综合改革，稳妥推进市人民医院改革试点。加强全科医生队伍建设，全面落实分级诊疗制度，基层首诊率达到50%以上。重视医德医风建设，构建和谐医患关系。继续实施妇女“两癌”筛查与救助工作。落实一对夫妇可生育两个孩子政策，促进人口长期均衡发展。

　　创新社会治理。深入推进平安铜川、法治铜川建设。认真实施“七五”普法规划，健全四级公共法律服务体系，增强全民法治意识。全面落实政府安全生产监管责任和企业主体责任，加强煤矿、交通、非煤矿山、建筑施工、油气管道、人员密集场所等重点领域的专项整治，深入开展“打非治违”专项行动，全面排查和化解安全生产隐患，坚决遏制重特大安全事故发生。依法从严保障食品药品安全。探索运用保险机制改进社会治理和公共服务方式，强化防灾减灾预警体系建设，提升应急救援能力。完善立体化社会治安防控体系，持续推进“大巡防”工作，不断增强人民群众安全感。严格落实信访工作责任，推进信访法治化建设，加强基层基础工作，构建矛盾纠纷多元化解机制，促进社会大局稳定。

　　四、以严和实的精神推进政府自身建设

　　围绕推进治理体系和治理能力现代化，加快法治政府、创新政府、廉洁政府和服务型政府建设，坚持以人民为中心的工作导向，对人民负责，为人民谋利益，接受人民监督。

　　严格依法行政。把政府工作纳入法治轨道，坚持以法治思维和法治方式履行职责，做到权力民赋、权依法使。全面实施法治政府建设五年规划，认真执行市人大及其常委会各项决议，自觉接受市人大法律监督、工作监督和市政协民主监督，虚心听取各民主党派、工商联、无党派人士和人民团体的意见。强化领导干部集体学法制度。围绕城乡建设与管理、环境保护、历史文化保护等领域，积极推进地方立法工作。完善行政决策合法性审查机制、重大决策终身责任追究制。公职人员要做尊法学法守法用法的模范，高悬法律的明镜，紧握法律的戒尺，始终在法治之下想问题、做决策、干事情。健全行政执法全过程记录制度，细化量化行政裁量基准，为权力行使定边界、划红线，做到规范公正文明执法。

　　扩大政务公开。依托“互联网+政务服务”平台，加大各领域办事公开力度，注重公开实效，让群众看得懂、能参与、好监督。改进直接面向企业和群众的公共服务，将实体政务大厅向网上办事大厅延伸。健全政府新闻发言人制度，全面及时准确发布政务信息，积极回应社会关切。坚持办好公民进政府、大学生到机关见习活动。完善公众代表列席市政府会议制度，做好网民留言办理和政务微博、微信发布，畅通公众意见反映和诉求听取渠道。开展代表、委员及网媒人士问政工作，以高效透明的服务体现政府为民谋利的诚意。加强经济宣传工作，把握主流和趋势，提振全社会加快发展的信心。

　　转变工作作风。按照“三严三实”的要求，从严从实转作风。严格遵守党的政治纪律和政治规矩，认真落实“八项规定”、国务院“约法三章”，坚持不懈纠正“四风”。建立健全厉行节约反对浪费长效机制，精简会议文件，严格节庆管理，从严控制行政成本，确保“三公”经费只减不增。加强公职人员教育管理，切实解决能力不足而“不能为”、动力不足而“不想为”、担当不足而“不敢为”等问题，让新思维、新理念落地生根、引领发展。强化责任担当和执纪问责，对外公开职责，对内闭环责任，实行“履责一单清”。完善绩效管理和考核评价机制，激励干事创业，严肃查处庸懒散行为。

　　加强廉政建设。认真贯彻《廉洁自律准则》和《纪律处分条例》，严格落实党风廉政主体责任和“一岗双责”。完善重点领域反腐倡廉制度建设，强化审计监督，从源头上预防和治理腐败。民心是最大的政治，正义是最强的力量。尊崇党章，敬畏国法，严守红线和底线。坚持无禁区、全覆盖、零容忍，坚决整治和查处侵害群众利益的不正之风和腐败分子，营造风清气正的政治生态。把严格管理干部和热情关心干部结合起来，形成心情舒畅、充满激情、层层负责、人人担当的良好工作氛围。

　　各位代表，追赶超越的目标鞭策着我们，全面建成小康社会的蓝图激励着我们。让我们紧密团结在以习近平同志为核心的党中央周围，在市委的坚强领导下，开拓创新，扎实工作，为建设转型铜川、美丽铜川、健康铜川、富裕铜川而努力奋斗！
